# Two mechanisms for direction selectivity in a model of the primate starburst amacrine cell

**DOI:** 10.1017/S0952523823000019

**Published:** 2023-05-23

**Authors:** Jiajia Wu, Yeon Jin Kim, Dennis M. Dacey, John B. Troy, Robert G. Smith

**Affiliations:** 1 Department of Biomedical Engineering, Northwestern University, Evanston, IL, USA; 2 Department of Biological Structure, Washington National Primate Research Center, University of Washington, Seattle, WA, USA; 3 Department of Neuroscience, University of Pennsylvania, Philadelphia, PA, USA

**Keywords:** Direction selectivity, starburst amacrine, primate retina, electrotonic propagation, modeling

## Abstract

In a recent study, visual signals were recorded for the first time in starburst amacrine cells of the macaque retina, and, as for mouse and rabbit, a directional bias observed in calcium signals was recorded from near the dendritic tips. Stimulus motion from the soma toward the tip generated a larger calcium signal than motion from the tip toward the soma. Two mechanisms affecting the spatiotemporal summation of excitatory postsynaptic currents have been proposed to contribute to directional signaling at the dendritic tips of starbursts: (1) a “morphological” mechanism in which electrotonic propagation of excitatory synaptic currents along a dendrite sums bipolar cell inputs at the dendritic tip preferentially for stimulus motion in the centrifugal direction; (2) a “space–time” mechanism that relies on differences in the time-courses of proximal and distal bipolar cell inputs to favor centrifugal stimulus motion. To explore the contributions of these two mechanisms in the primate, we developed a realistic computational model based on connectomic reconstruction of a macaque starburst cell and the distribution of its synaptic inputs from sustained and transient bipolar cell types. Our model suggests that both mechanisms can initiate direction selectivity in starburst dendrites, but their contributions differ depending on the spatiotemporal properties of the stimulus. Specifically, the morphological mechanism dominates when small visual objects are moving at high velocities, and the space–time mechanism contributes most for large visual objects moving at low velocities.

## Introduction

**Figure 1.**
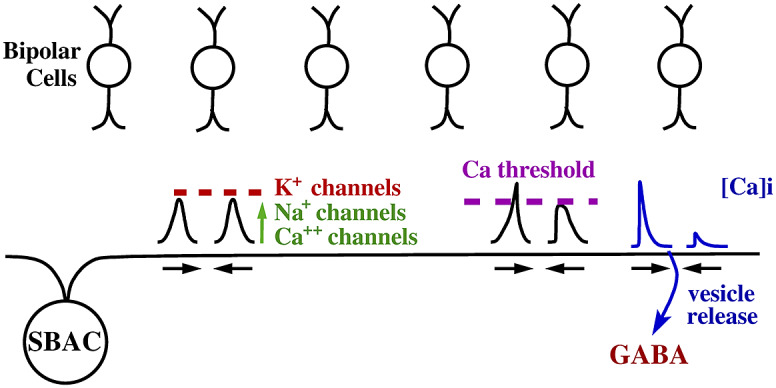
Schematic diagram to illustrate the basic biophysical features of a starburst amacrine (SBAC) dendrite. EPSPs (black peaked traces) evoked by a bar moving across the entire dendritic tree (black horizontal arrows indicate direction) at proximal locations along the dendrite are similar in amplitude, but at distal locations are larger in the centrifugal (outward) direction than the centripetal (inward) direction. K^+^ channels (red), present in the soma and proximal dendrites of the real cell, are thought to limit depolarization (red dashed line) to less than −20 mV (Ozaita et al., 2004). Na^+^ and Ca^++^ channels (green) are also present in the dendrites, and thought to amplify (green arrow) EPSPs highly nonlinearly above a voltage threshold (Ca threshold, magenta dashed line) to generate all-or-none Ca^++^ events (large blue peak) from motion in the centrifugal direction. Motion in the centripetal direction does not reach the voltage threshold for strong activation of Ca channels, supporting a large directional difference in [Ca]i (blue peaked traces). Varicosities containing neurotransmitter vesicles are located at the distal end of the dendrite, where rising Ca^++^ levels (blue peak) during centrifugal motion cause neurotransmitter release (blue arrow), indicated here as GABA. Notably, the directional difference in EPSPs and [Ca]i levels is thought to be greatest at the distal region where Ca^++^ entry triggers neurotransmitter release (Koren et al., 2017).

Studies in rabbit and mouse have demonstrated that directional-selective signaling originates in the retina with the starburst amacrine cell, an inner retinal interneuron whose dendrites extend radially from its soma and branch several times to generate a distinctive “starburst” morphology (Famiglietti, 1983, 1991; Miller & Bloomfield, 1983; Tauchi & Masland, 1984; Vaney, 1984). When starburst amacrines are selectively ablated from the retina of the mouse and rabbit, the animals lose both the optokinetic reflex and the direction-selective response of ganglion cells that receive inhibitory input from starburst cells (Yoshida et al., 2001; Amthor et al., 2002), implying an important role for the starburst amacrine in retinal computation of motion direction. Individual starburst dendrites serve as computational subunits that receive synaptic input from an array of depolarizing bipolar cells. Each dendrite responds by depolarizing more to motion from soma to dendritic periphery (centrifugal) where transmitter is released than in the opposing (centripetal) direction (Euler et al., 2002; Hausselt et al., 2007; Koren et al., 2017; Kim et al., 2022; Fig. 1). These dendritic outputs are then selectively sampled by postsynaptic retinal ganglion cells to drive null direction inhibition and direction selectivity (DS; Fried et al., 2002, 2005; Münch & Werblin, 2006; Briggman et al., 2011; Yonehara et al., 2016). The presence of such amacrine and ganglion cells in mouse and rabbit retina have been known for decades (Barlow et al., 1964; Sun et al., 2006) and recently their counterparts have been demonstrated physiologically in the macaque (Kim et al., 2022).

Kim et al. (2022) provided a description of the macaque ON starburst morphology and a connectomic analysis of its bipolar cell inputs. Like starbursts in other species, macaque starburst dendrites emanating from the soma become extremely thin as they successively branch several times, and then enlarge into varicosities near the distal tips. ON midget bipolar cells provide input to the full dendritic tree while input from the DB4/5 bipolar cells (Tsukamoto & Omi, 2016)^1^ is restricted to distal regions. As for recordings in rabbit and mouse, somatic recordings from macaque starburst amacrine cells show large directional differences in their response to centrifugal *versus* centripetal motion when stimulated with radial gratings (Euler et al., 2002; Hausselt et al., 2007; Kim et al., 2022). Calcium imaging of individual macaque starburst amacrine dendrites reveals a similar preference for centrifugal motion (Kim et al., 2022).

There are competing theories about what microstructural elements account for the generation of directional signaling in starburst dendrites. One proposes that the electrotonic summation of bipolar cell input in the dendrites results in a directional signal with larger depolarizations generated at distal synaptic varicosities for centrifugal than for centripetal image motion (Rall, 1964; Tukker et al., 2004). We refer to this as the “morphological” mechanism because it is closely linked to the dendritic morphology, including the branching pattern and progression of dendritic diameter from the soma outwards to the medial and distal dendrites (Tukker et al., 2004). A second theory proposes that spatially differential synaptic inputs from sustained and transient bipolar cells to the dendrites generate a directional signal (Kim, 2014; Fransen & Borghuis, 2017; Srivastava et al., 2022). This has come to be known as the “space–time” mechanism (Kim, 2014). Since mouse and macaque are known to possess bipolar cells with both sustained and transient response kinetics, and their starburst amacrine cells are similar in morphology, we reasoned that very likely both morphological and space–time mechanisms could function in these two species.

In the primate visual system, two dominant parallel pathways transmit sustained and transient signals *via* the lateral geniculate nucleus to primary visual cortex (De Monasterio & Gouras, 1975; Lee et al., 2010; Dacey et al., 2014; Wool et al., 2018). The midget pathway is more sustained and involves midget bipolars, whereas the parasol pathway involves DB4/5 bipolars which have a transient response (Puthussery et al., 2013). Kim et al. (2022) showed surprisingly that ON starburst amacrine cells receive synaptic contacts from both the midget-sustained and DB-transient pathways. The midget bipolars make synaptic contacts across the entire starburst dendritic arbor, and DB4/5 bipolar cells make synaptic contacts in the distal dendritic regions, consistent with providing a basis for the space–time mechanism. We constructed computer models that could include or omit the transient DB4/5 bipolar cell. This allowed us to gauge the relative contributions of the bipolar cell-based “space–time” mechanism *versus* the “morphological” based mechanism to directional signaling in the dendrites of macaque starburst cells and to determine to what range of stimuli each might be best attuned. We also constructed similar models using known mouse morphology and connectomics, including bipolar cells with sustained and transient response kinetics, to gauge their relative contributions and to compare with results from the macaque models.

## Materials and methods

### Starburst morphology

**Figure 2.**
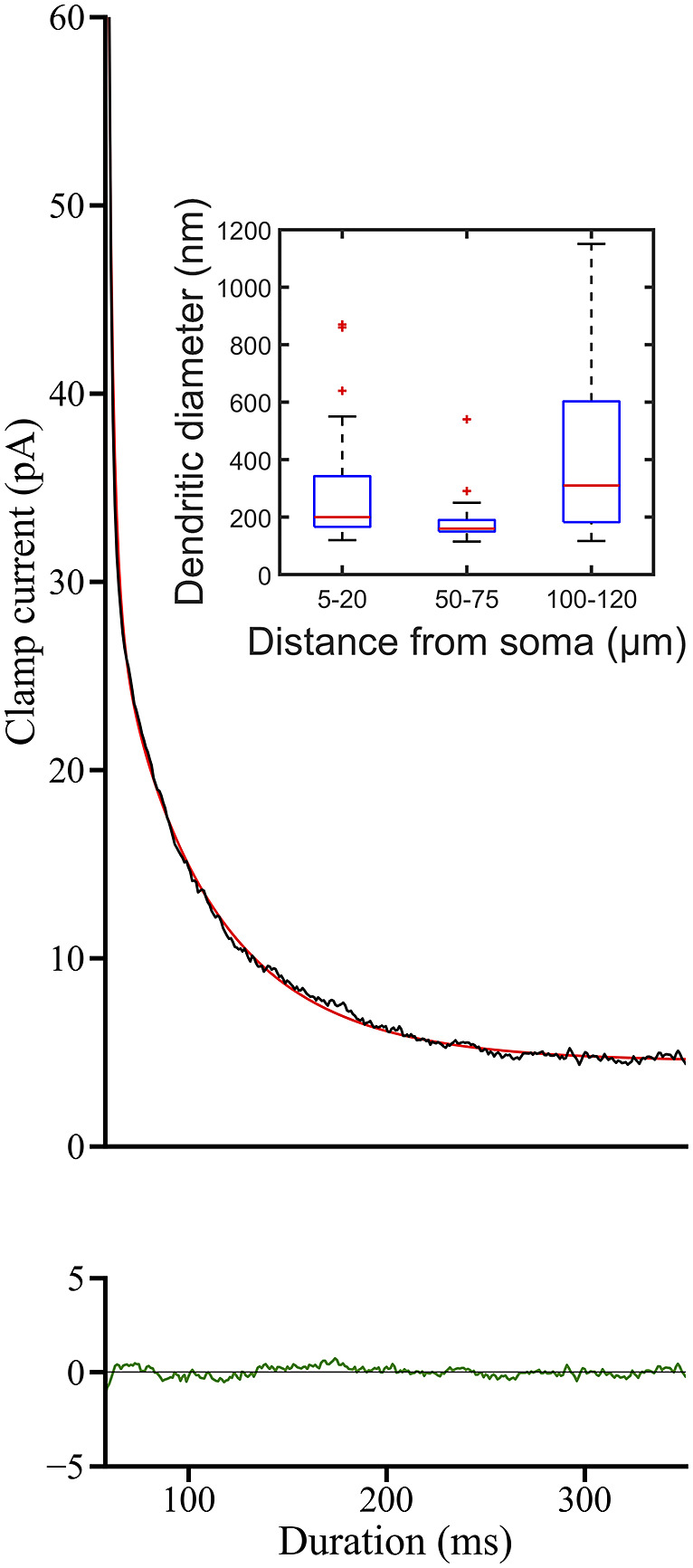
Plot showing the current charging curve from somatic voltage clamp in a macaque starburst amacrine cell, which was least-squares fitted to estimate the diameters of starburst dendritic regions. Black trace is the averaged current charging curve of the recorded starburst cell (*V*_step_ = 5 mV). Red trace is the optimized model fit, based on proximal dendrites of 0.3 *μ*m, medial dendrites of 0.2 *μ*m, and distal dendrites of 0.4 *μ*m. Below, green, plot of difference between red and black traces. Inset, plots of proximal (5–20 *μ*m), medial (50–75 *μ*m), and distal (100–120 *μ*m) dendrite diameters measured in original EM serial sections from Kim et al. (2022). The central red line indicates the median, and the top and bottom edges of the box indicate the 75th and 25th percentiles, respectively. The dashed whiskers extend to the most extreme data points not considered outliers, and the outliers are plotted individually with a red “+” symbol.

The model of a macaque ON starburst amacrine cell was built in the Neuron-C simulator (Smith, 1992). The cell’s morphology was derived from the tracing of a 2-photon image of a macaque ON starburst physiologically identified and intracellularly labeled in the retina periphery (Kim et al., 2022). The overall width of the starburst dendritic tree was ~250 *μ*m, consistent with the morphology of starbursts from the periphery of the macaque retina. The cell was modeled with 400 ~ 700 compartments, each spanning ~0.02 length constants. Length (*λ*) and time constants (*τ*) were determined by the axial resistivity, the membrane resistivity, and the membrane capacitance following standard practice (Rall & Agmon-Snir, 1989). The thickness of a dendrite was allowed to change along its length. Somatic current charging curves were recorded from macaque starbursts, and a model of the charging currents in a starburst without synaptic input was least-squared fitted to estimate the dendritic section (proximal, medial, distal) diameters, as described in Kim et al. (2022) (Fig. 2). The model contained 5 free parameters: proximal, medial and distal dendritic diameter, membrane resistivity for the cell soma, and membrane resistivity for cell dendrites (Table 1). Axial resistivity (Ri) was manually set to 100 Ωcm because it was correlated with the dendritic diameters. Membrane capacitance was set to 1 *μ*F/cm^2^. Electrode resistance was set manually to 20 mΩ. The soma diameter was manually set (9 *μ*m) because it inversely correlated with the proximal dendritic diameter. Synaptic inputs and voltage-gated ion channels were omitted. The best-fit dendritic diameter parameters were: proximal 0.3 *μ*m, medial 0.2 *μ*m, and distal 0.4 *μ*m. The real dendritic diameters, measured in the serial electron microscope sections from the Kim et al. (2022) study, were proximal 0.2–0.3 *μ*m, medial 0.15–0.2 *μ*m, and distal 0.4–0.45 *μ*m (see Fig. 2, inset). The fitted diameter factors qualitatively recapitulated the dendrite thicknesses observed in the original connectomic reconstructions—proximal: intermediate; medial: very thin; distal: thick with varicosities, and were similar to the dendritic diameters of mouse starbursts (Ext. Data Fig. 7 in Ding et al., 2016).

**Table 1.** The best-fitted parameters for the current charging curves from voltage clamp of a macaque starburst amacrine cell

	Parameters	Units	Values
SBAC model	Membrane resistivity (Rm) of soma	kΩcm^2^	22
	Membrane resistivity (Rm) of dendrites	kΩcm^2^	41
	Proximal dendritic diameter	*μ*m	0.37
	Medial dendritic diameter	*μ*m	0.19
	Distal dendritic diameter	*μ*m	0.4

### Location and synaptic connections of bipolar cells

**Figure 3.**
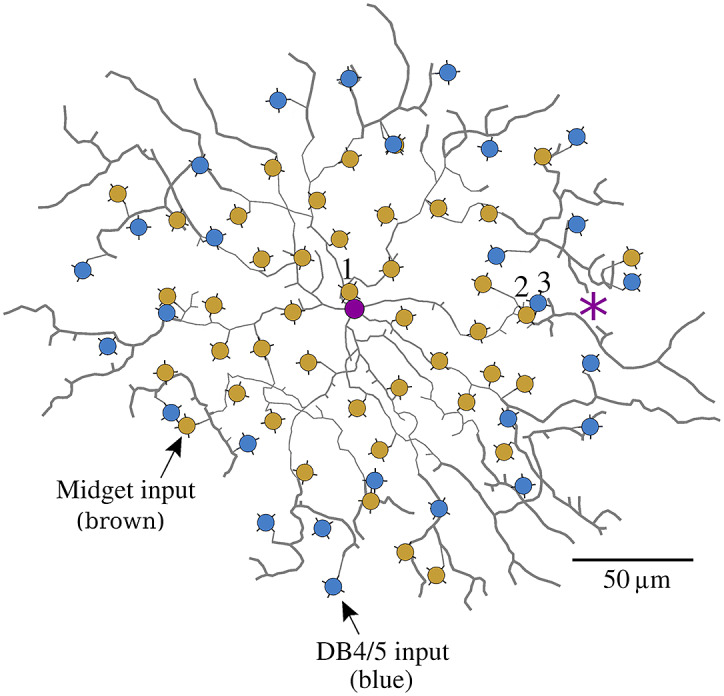
Diagram of the model starburst dendritic tree showing the locations of the soma (dark magenta), and midget (brown) and DB4/5 (blue) bipolar cell inputs. Later figures refer to the bipolar cells labeled 1, 2, and 3, and the purple asterisk at right which marks the location of voltage and calcium signal recordings. This view is representative of 30 models in which the starburst was randomly rotated and bipolar cell locations were randomly selected (see Materials and methods).

Following connectomic results for the macaque ON starburst amacrine cell, an array of ON midget (sustained) bipolar cells was arranged to provide input to the full dendritic tree while input from an array of DB4/5 (transient) bipolar cells was restricted to distal dendritic regions (Fig. 3). To test the role of the morphological mechanism, the transient DB4/5 bipolars were replaced in the model with sustained midget bipolars, keeping the total number of bipolar cell inputs unchanged. Bipolar cell locations were generated to simulate realistic cellular arrays (density, midget 1900 cells/mm^2^; DB4/5 900 cells/mm^2^; regularity index 8; as generated in Kim et al., 2022) which recapitulated the connectomic results of ON midget and DB4/5 bipolars (in a limited starburst reconstruction, midget *n* = 25; DB4/5 *n* = 23; Kim et al., 2022). The resulting cellular arrays had a total of 77 ± 4.9 s.d. bipolar cells (midget 41 ± 4.2 s.d.; DB4/5 35 ± 2.1 s.d.; *n* = 30). A synaptic contact was made from a bipolar cell when its location was within a criterion distance (10 *μ*m) of a starburst dendrite. This resulted in 88 (± 5.3 s.d.) bipolar cell contacts onto the starburst dendritic arbor. The conductance was set relatively low (10 ~ 20 pS) to keep the resulting EPSP peak amplitudes below −50 mV, limiting any potential saturation effect.

### Biophysical mechanisms

The bipolar cell morphology included a soma, an axon (length 20 *μ*m, dia 0.3 *μ*m), and four axon terminal branches (length 5 *μ*m, dia 0.2 *μ*m). The light-evoked responses of midget bipolar cells were modeled with slowly inactivating calcium channels and a calcium pump to produce a sustained conductance generated with a readily releasable vesicle pool (15 ves.) with a relatively fast replenishment rate (1000/s). Transient responses of DB4/5 bipolar cells were modeled by including voltage-gated sodium channels (~2500 mS/cm^2^ NaV1.1, Puthussery et al., 2013) and slowly inatcivating potassium channels (~1 mS/cm^2^ of Kv1.1 and Kv3) in the bipolar cell axon terminal, and including the same size vesicle pool (15) with a slow replenishment rate (200/s). The Na channels generated a slow action potential (10–20 ms) on the leading edge of the stimulus that activated the calcium channels to drive vesicle release. The rationale for including the readily releasable vesicle pool and the sodium and potassium channels was to generate qualitatively realistic bipolar responses, not to test specific features of neurotransmitter release in the bipolar cells. In some models, slowly inactivating N/P/Q-type calcium channels were included in the starburst cell (soma and proximal dendrites, 0.2 mS/cm^2^; medial dendrites, 3.5 mS/cm^2^; distal dendrites, 7.5 mS/cm^2^) (Figs. 1 and 10B,C). The duration of calcium events was controlled by a calcium pump (*V*_max_ = 0.2–0.7 *μ*A/cm^2^, *K_m_* = 30 *μ*M), setting a fall time of 300–500 ms. We did not attempt to closely calibrate the exact details of these biophysical mechanisms.

### Stimulus and recording site

The entire starburst cell was stimulated with a light bar moving across a field larger than the dendritic tree first from left to right and then from right to left. The bar was 500 *μ*m long (orthogonal to the direction of motion) so it extended over the entire starburst cell. Bar widths (in the direction of motion) ranged from 50 to 500 *μ*m, and bar velocities ranged from 100 to 10,000 *μ*m/s. This range of velocities in the macaque eye translates to 0.5 to 50 deg/s (200 *μ*m/deg. visual angle), which is generally considered to be a relevant range for behavioral/psychophysical studies of primates (see section “Relation to behavioral performance”). Stimulation of the bipolar cells was accomplished by clamping their soma in accordance with the spatial stimulus using a “background intensity” of −56 mV and a typical depolarizing “contrast” of 7 mV. The stimulus included a bipolar receptive field (RF) with Gaussian optical blur functions (RF center dia. 30 *μ*m, RF surround dia. 120 *μ*m, surround/center integrated weight typically 0.7, surround-center delay 10 ms; Kim et al., 2022). The RF surround subtracted from the center response which, for a moving bar stimulus, generated two transients in the center response. Simulated voltage and, for some simulations, calcium responses, were measured near the starburst dendritic tips (purple asterisk in Fig. 3) because that is thought to be the location where synaptic vesicles containing GABA are released onto postsynaptic neurons. Other recording sites in the distal tip region produced almost identical simulated response amplitudes and waveshapes because the diameter of the dendritic tips was relatively large (0.4 *μ*m) which minimized electrotonic decay within the distal region. Recording sites more proximal to the soma gave qualitatively similar voltage waveshapes to the distal ones, but with a reduced directional difference, and simulated somatic voltage recordings had a directional difference near zero.

### Effect of surround delay and weight

Our models were run with bipolar cell RF surrounds with a delay of 10 ms. This delay is similar to RF surround delays reported in previous work. We also ran models with zero delay, as well as 20 and 40 ms delays, and found that the bipolar cell response waveshape was qualitatively similar. The main effect of larger surround delays was to amplify the initial transient in the bipolar response. This initial transient was further emphasized by the slowly inactivating calcium channels in the bipolar cell synaptic release mechanism, along with a readily releasable vesicle pool. In the transient DB4/5 bipolars, sodium and potassium channels generated a 10–20 ms action potential which caused a large transient in neurotransmitter release (Puthussery et al., 2013). For large, slow stimuli, the bipolar surround relative weight of 0.7 generated a “trough” between initial and secondary peaks in the bipolar response waveshape. Weaker surround weights (0.5, 0.6) generated a shallower trough and effectively caused a larger center response. These mechanisms included in the bipolar cells were intended only to generate plausible responses. Their parameter sets were not explored exhaustively nor calibrated against real data, as the exact waveshapes of bipolar cells evoked by moving bars of different widths are not precisely known.

### Rationale for the use of moving bar stimuli

We utilized linear moving stimuli that evoked bipolar cell responses over the entire starburst dendritic arbor and our simulated recordings were made near the dendritic tips (purple asterisk in Fig. 3). In contrast, somatic recordings from starburst amacrines have often been made with radially moving stimuli, or stimuli masked near the soma (Euler et al., 2002; Hausselt et al., 2007; Oesch & Taylor, 2010; Fransen & Borghuis, 2017; Kim et al., 2022). Although such stimuli evoke strong somatic directional responses in starburst amacrines, recent studies provide evidence that this type of “appear-then-move” stimulus generates motion sensitivity in the presynaptic bipolar cells (Gaynes et al., 2022; Strauss et al., 2022). When this type of radial or masked stimulus first appears, it evokes a strong response to motion away from the bipolar RF center when the bipolar’s surround response is delayed. The effect is especially evident in somatic recordings with radially moving stimuli centered over the starburst soma, because the radial stimulus evokes a strong motion sensitivity in proximal bipolar cells. However, stimuli that pass over the entire bipolar cell (and starburst) RF do not evoke this type of motion sensitivity because stimuli that pass into and out of the bipolar RF center evoke symmetric responses (Gaynes et al., 2022; Kim et al., 2022; Strauss et al., 2022). Therefore, for simplicity in the comparison between the morphological and space–time mechanisms, we chose to omit radial stimuli that would generate motion sensitivity originating in the bipolar cell from its center-surround RF.

### Randomized models and statistics

Multiple instances of each model (*n* = 30) were run with randomly specified rotations of the starburst and with randomized bipolar cell locations. Simulations took 2–200 min on 3200 MHz servers, comprising a total of more than 200,000 models. The mean direction-selective index [DSI = (Pref peak resp − Null peak resp)/(Pref peak resp)] and its standard error were computed from these randomly generated models. The DSI values computed for the models with midget and midget + DB4/5 bipolars were compared using a standard two-tailed paired *t*-test to produce *P*-values that represented the probability that there was no difference between the mean DSIs (see Table 2). The *P*-value computations were run with the Python language and separately with the Neuron-C simulation language (Smith, 1992).

### Model of mouse starburst

**Figure 4.**
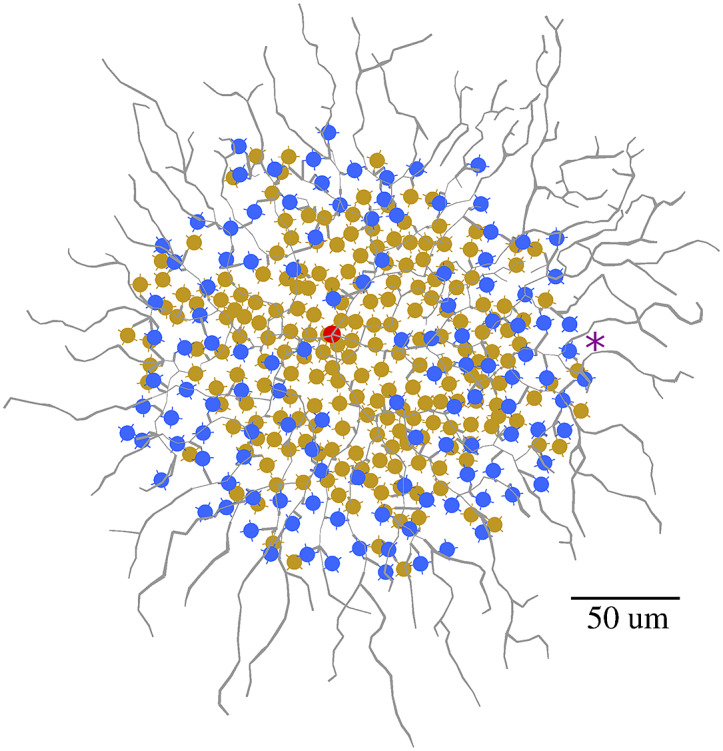
Diagram of the mouse starburst dendritic tree showing the soma (red) and locations of sustained (brown) and transient (blue) bipolar cell inputs. The starburst morphology was taken from Ding et al. (2016). The purple asterisk at the right indicates the recording location. This view is representative of 30 models in which the starburst was randomly rotated, and bipolar cell locations were randomly selected (see Materials and methods).

In order to derive more intuition about how DSI is related to the model parameters, we ran comparison models using mouse starburst morphology and connectomics derived from Ding et al. (2016). The dendritic arbor of the mouse starburst was similar in extent to the monkey (250 *μ*m) but the bipolar cell inputs to the model starburst were limited to the inner 2/3 of the dendritic radial length (Fig. 4), and the number of modeled bipolar cells providing input was ~4-fold greater in random sampling (total 339 ± 10.1 s.d., sustained 215 ± 7.9 s.d.; transient 124 ± 5.7 s.d.; sustained 10,000/mm^2^; transient 4,600/mm^2^; *n* = 30). Because the model of the mouse starburst received ~4-fold more bipolar inputs, their postsynaptic conductance was reduced by 70% from the macaque model (to 3–7 pS) to maintain the EPSP amplitude below −50 mV.

## Results

### Evaluating the space–time mechanism

**Figure 5.**
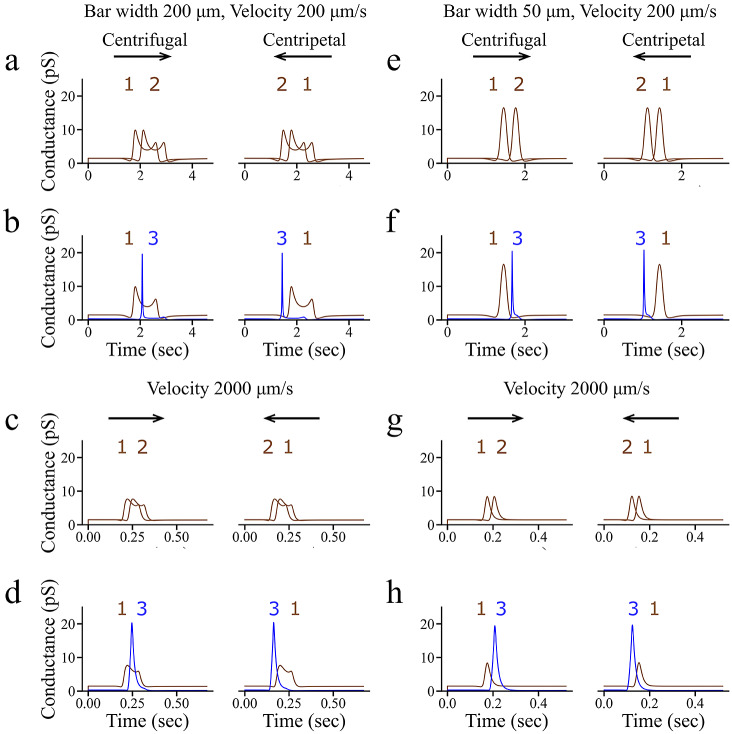
The space–time mechanism functions mainly with large object sizes. (A–H) Plots showing the conductance changes induced in the starburst dendrite from bipolar cell activation. Conductance changes labeled 1, 2, and 3 correspond to activation of the bipolar cells so labeled in Fig. 3. (A) Plots of conductance changes from a model in which the DB4/5 bipolars are replaced with midget bipolars (brown), in response to a bar of 200 *μ*m width moving in centrifugal (left column) and centripetal (right column) directions with a velocity of 200 *μ*m/s. The modeled midget bipolar cells’ excitatory response to a bar moving from left to right begins with an initial transient as the bar enters the RF center from the left. This is followed by a trough when the leading edge of the bar exits the center and encroaches on the right-hand flank of the surround. The trough remains until the trailing edge of the bar exits the left-hand flank of the surround and reaches the left-hand edge of the center. A second transient similar to the one at the start of the response occurs as the stimulus replays in reverse the initial pattern of stimulation. In this model, proximal (1) and distal (2) midget bipolar-induced conductances overlap symmetrically between centrifugal and centripetal directions, thus generating no directional preference. (B) Plots of conductance changes from a model including DB4/5 bipolars (blue). In this model, proximal midget (1, brown) and distal DB4/5 (3, blue) bipolar-induced conductances overlap asymmetrically, because the bar width (200 *μ*m) is greater than the distance between proximal and distal bipolar inputs, thus generating, after postsynaptic summation, a directional preference for stimulation in the centrifugal direction. (C,D) Plots from the same models as for (A,B) but for a bar of 200 *μ*m width moving at 2000 *μ*m/s. (E,F) Plots from the same models as (A,B), but for a bar of 50 *μ*m width moving at 200 *μ*m/s. (G,H) Plots from the same models as (A,B), but for a bar of 50 *μ*m width moving at 2000 *μ*m/s. In models with DB4/5 bipolars and a bar width of 50 *μ*m (F,H), the conductances show less overlap (than B,D) because the bar width is smaller than the distance between proximal and distal bipolar inputs, so less directional signal is present in the bipolar inputs, illustrating that the contribution of the space–time mechanism to directional signaling is not activated by narrow bars.

**Figure 6.**
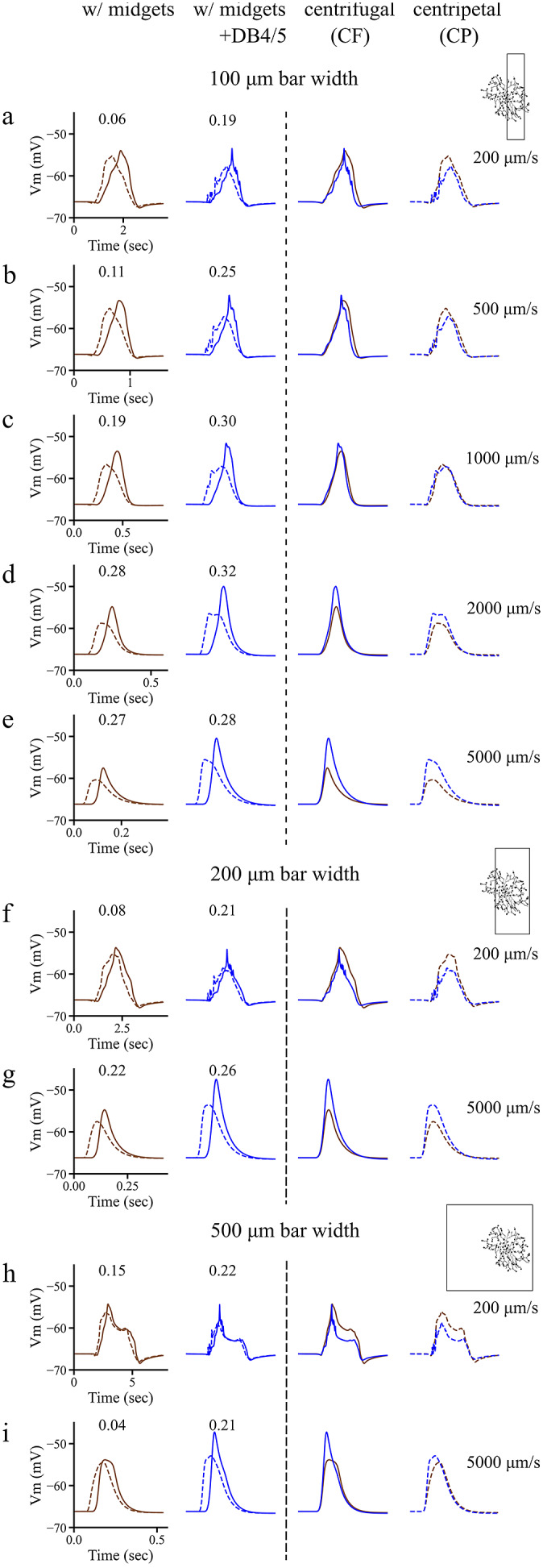
Comparison of the relative contributions of the morphological and space–time mechanisms to direction-selective index (DSI) for different velocities. (left 2 columns) Responses to CF and CP motion are superimposed to facilitate comparison of waveshape, peak amplitude, and DSI. (A–E) Plots of distal (purple asterisk in Fig. 3) starburst dendritic voltage responses (solid traces, CF motion; dashed, CP motion; brown, midget bipolars only; blue, midget, and DB4/5 bipolars) evoked by a bar of 100 *μ*m width moving at different velocities (200, 500, 1000, 2000, and 5000 *μ*m/s). Average DSIs (*n* = 30) are included in each panel, but the plotted waveshapes are representative examples and are not averaged. (Right 2 columns) Plots of the same responses as in the left 2 columns, but with midget and midget + DB4/5 responses superimposed to facilitate intuition about the effect of DB4/5 transient responses on waveshape. (F,G) Plots of distal starburst dendritic voltage responses evoked by a bar of 200 *μ*m moving at 200 and 5000 *μ*m/s. (H,I) Plots of distal dendritic voltage responses evoked by a bar of 500 *μ*m moving at 200 and 5000 *μ*m/s. (D,E,G,I) With high-velocity stimuli, the morphological mechanism generates DS. (A–C,F,H) With low velocity and wide bars, both the morphological and space–time mechanisms generate DS. Thirty instances of each model were run for randomly specified starburst rotations and bipolar cell locations to produce a mean DSI, which is noted above the left-hand traces in each case. Insets at right show the bar width; the position of the bar in each inset is a snapshot of the stimulus at one point along the *x*-axis, so the “time” position differs for each inset. CF and CP motion refers to the relationship between the recording site and the soma. In both cases, the stimulus traverses the full dendritic tree. For CF motion, the leading edge of the stimulus crosses the soma before the recording site. For CP motion, the leading edge of the stimulus crosses the recording site before the soma. The standard errors are shown in Fig. 8 and Table 2. DSI increases with velocity up to 2000 *μ*m/s for both models, and the addition of DB4/5 bipolar inputs increases DSI for all stimulus conditions tested here, with the effect most pronounced at low velocity (see section “Interactions with waveshape evoked by large objects”; Fig. 8 and Table 2).

To investigate how differences in response time-course between midget and DB4/5 bipolar cells might contribute to directional signaling, we simulated either sustained (midget) or sustained + transient (midget + DB4/5) bipolar-induced conductance changes in starburst dendrites. We started with a bar 200 *μ*m wide moving in both centrifugal (CF) and centripetal (CP) directions at a velocity of 200 *μ*m/s. For a model with midget bipolar cells alone providing input to the starburst, the overlap of conductance changes induced for proximal (bipolar cell labeled 1 in Fig. 3) and distal (bipolar cell labeled 2 in Fig. 3) dendritic locations showed no difference for centrifugal and centripetal directions of stimulus motion (Fig. 5A). When DB4/5 bipolar cells were substituted for some of the distal midget bipolar cells (Fig. 5B), the proximal midget (brown trace) and distal DB4/5 (bipolar cell labeled 3 in Fig. 3; blue trace) bipolar-induced conductances in the starburst dendrite for centrifugal and centripetal stimulus motion overlapped asymmetrically. A similar result was produced with the same 200 *μ*m bar width but a higher velocity (2000 *μ*m/s); even though the response was only ~0.1 s in duration, the sustained proximal and transient distal bipolar conductance changes overlapped asymmetrically to generate a centrifugal preference (Fig. 5C,D). This asymmetric bipolar conductance input when summed as EPSPs in the starburst dendrite evoked a directional preference, with a larger summed amplitude in the centrifugal direction. When the visual stimulus was a narrower bar (50 *μ*m) moving at low velocity (200 *μ*m/s) or higher velocity (2000 *μ*m/s), the bipolar inputs did not sum temporally, as conductances produced by both midget and DB4/5 bipolar cells decay quickly after the stimulus passes (Fig. 5E–H), so the direction preference from spatially differential bipolar cell inputs was lost. Postsynaptic V_m_ recordings also show the summed overlap between proximal and distal bipolar inputs, but reflect the summation of all the bipolar inputs on the dendrite (Fig. 6). At higher velocities, (2000–10,000 *μ*m/s) wide bars evoked short postsynaptic *V_m_* responses at the distal recording site with directional differences that reflected electrotonic propagation within the dendrite and also temporal summation of proximal and distal bipolar inputs (Fig. 6D,E,G,I).

**Figure 7.**
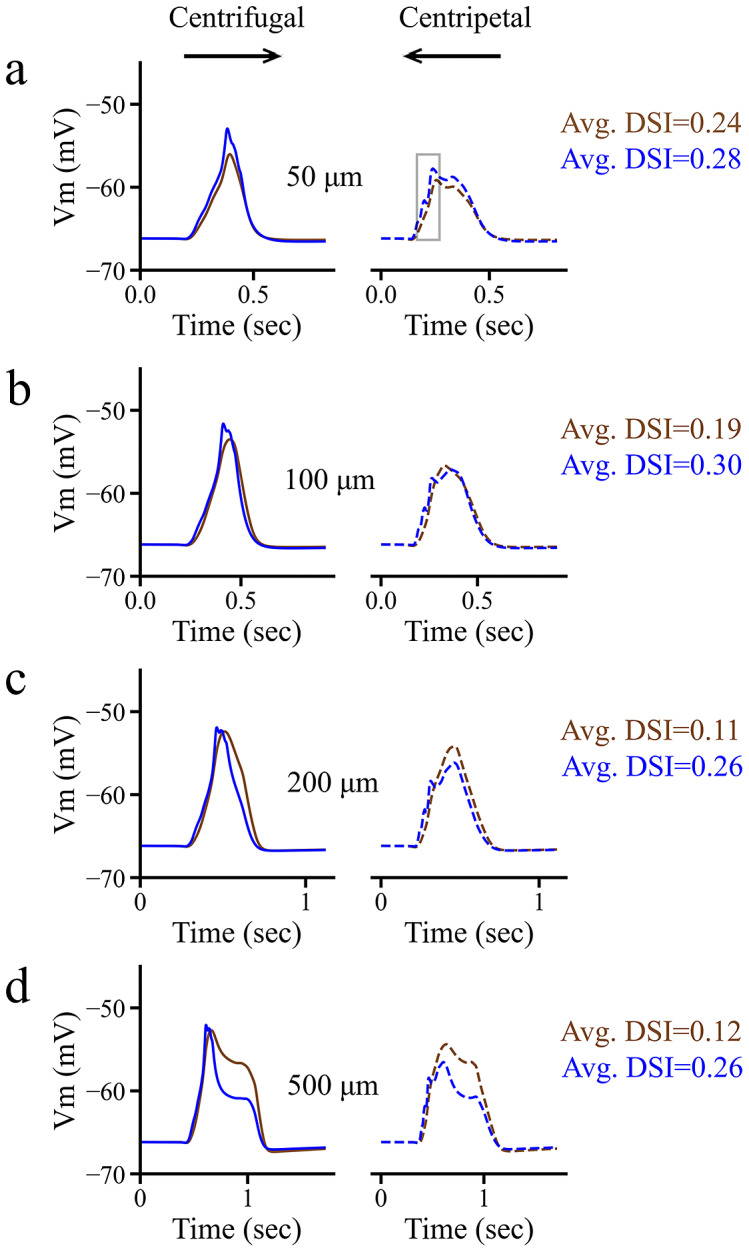
The morphological mechanism provides the largest component of DS evoked by small objects moving at high velocity. Comparison of the relative contributions of the morphological and space–time mechanisms to DS for different stimulus sizes. Representative plots of distal starburst dendritic voltage responses evoked by bars of different sizes (A) 50 *μ*m, (B) 100 *μ*m, (C) 200 *μ*m, and (D) 500 *μ*m moving at 1000 *μ*m/s (solid traces, CF motion; dashed, CP motion; brown, midget bipolars only; blue, midget and DB4/5 bipolars as shown in Fig. 3). Average DSI (*n* = 30) tends to increase as the object size decreases for both models, and for most stimuli the inclusion of DB4/5 inputs increases DSI over the model based purely on midget bipolar drive. The gray rectangle in (A) highlights the initial transient in the centripetal response evoked by a 50 *μ*m bar that originated in a distal transient DB4/5 bipolar cell. Larger bars (B–D) evoke the same transient, but it is superseded by a delayed response peak.

Since the directional preference for bipolar-induced conductance changes in starburst dendrites depends on stimulus conditions, as shown, we examined the voltage responses recorded from a distal dendritic location (purple asterisk in Fig. 3) for bars with a range of velocities (Fig. 6) and bar widths (Fig. 7). Two models were considered, the first where only midget bipolars provide drive to the starburst and the second where DB4/5 bipolar cells provide a large fraction of the input to distal dendritic regions. Inclusion of DB4/5 bipolar inputs (blue) increased the DSI of starburst dendritic voltage responses over the model with only midget bipolar inputs (brown) for all stimuli shown in Figs. 5 and 6, consistent with the directional preference for bipolar-induced conductance changes. With a large stimulus (bar width 200 or 500 *μ*m; greater than the distance between proximal and distal bipolar inputs) moving at low velocity (200 *μ*m/s; Fig. 6A), the small transient evoked by DB4/5 bipolars overlapped with the peak response evoked by midget bipolars in the centrifugal direction but not in the centripetal direction of stimulus motion because of a temporal difference. This voltage peak from the DB4/5 bipolars evoked a greater DSI in starburst voltage responses. For bar widths less than the distance between proximal and distal bipolar inputs, the number of midget bipolars with responses that overlapped the distal bipolars became smaller. For example, with a bar width of 100 *μ*m, the response from midget bipolars located midway to the distal bipolars overlapped the small transient from DB4/5 bipolars, but with a bar width of 50 *μ*m fewer midget responses overlapped those of the distal bipolars.

### Evaluating the morphological mechanism

**Figure 8.**
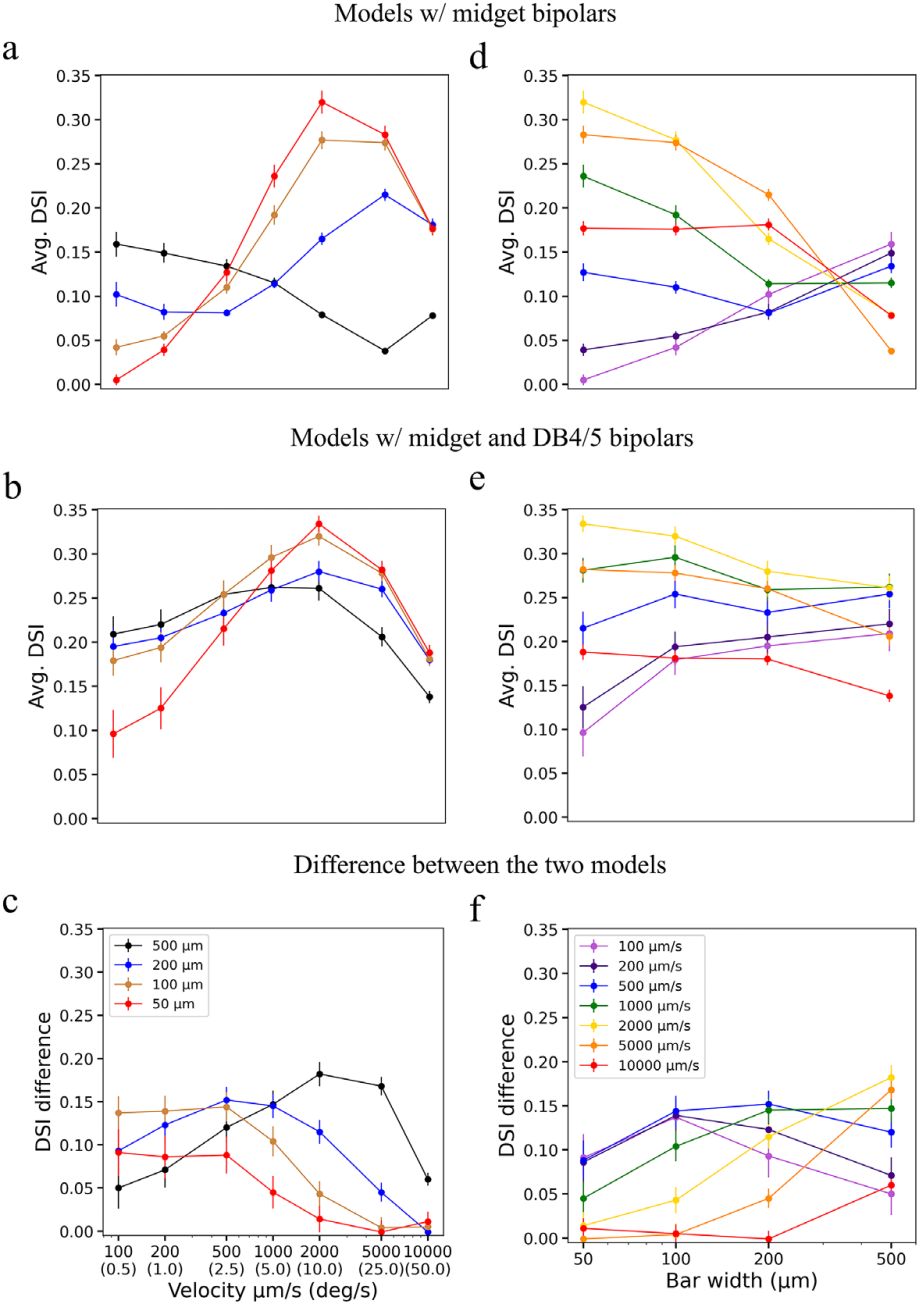
The morphological mechanism for DS functions well for small objects and fast velocities, and the “space–time” mechanism functions well for large objects and low velocities (*P* < 0.005; see Table 2). (A–C) Summary plots of average DSI *versus* velocity for (A) midget models and (B) midget + DB4/5 models, and (C) the difference (B − A). DSI for 50 (red trace) and 100 *μ*m bars (brown trace) is robust and similar with and without DB4/5 transient bipolar cells at velocities of 1000 *μ*m/s or more. DSI for 200 and 500 *μ*m bars is increased at low velocities by DB4/5 transient bipolar cells. The velocities in deg/s are calculated for the macaque retina (1 deg = 200 *μ*m). Mouse velocities in deg/s are ~7-fold higher. (D–F) Summary plots of average DSI *versus* bar width for (D) midget and (E) midget + DB4/5 models, and (F) the difference (D,E). Plots (A) and (D) show the effect of the morphological mechanism. Difference plots (C,F) show the effect of the “space–time” mechanism. Vertical bars indicate standard errors (*n* = 30). The DSI trend in models with only midget bipolars and 500 *μ*m bar width in (A) (black trace) differs from the other bar widths because of the different waveshape evoked by the 500 *μ*m bar (see Fig. 6 and section “Interactions with waveshape evoked by large objects”).

The voltage responses from the model depended on temporal summation of excitatory synaptic input due to electrotonic propagation that was affected by different velocities and stimulus sizes. The model with only midget bipolars generated larger voltage responses than the model with midget and DB4/5 bipolars in both directions of stimulus motion (Figs. 6 and 7), because the more sustained responses of the midget bipolars produced greater integrated conductance changes than the more transient DB4/5 bipolars. The starburst response amplitude increased with the size and velocity of the visual stimuli as more bipolar inputs were temporally summed. At the distal recording site, temporal summation of EPSPs from proximal and distal bipolar cells was maximal for centrifugal motion when the stimulus velocity equaled the electrotonic propagation velocity. For the parameter set used in these models, this optimal velocity was ~2000 *μ*m/s (Figs. 6 and 8). With small (50, 100 *μ*m bar width) stimuli moving at high velocity (1000–10,000 *μ*m/s; 5–50 deg/s), the responses to centrifugal motion were maximal (Fig. 6C–E). In contrast, in response to centripetal motion at the same velocities (1000–10,000 *μ*m/s), the distal bipolar inputs evoked a rapidly rising EPSP at the distal recording site, but proximal bipolar inputs were delayed and did not temporally sum with the distal inputs, generating a wider but lower response at the distal recording site. This effect increased the directional difference and DSI for the higher velocities (Figs. 6 and 8).

### Interactions with waveshape evoked by large objects

With larger bars (200 and 500 *μ*m) and low velocities (100 and 200 *μ*m/s) the stimulus bar generated 2 transients as it entered and exited the bipolar cell RF center (Fig. 5B). These transients interacted in different ways, depending on the velocity and size of the bar and thus the bipolar cell response duration. At 200 *μ*m/s, for the models with only midget bipolars and a 500 *μ*m bar (Fig. 6H, brown traces), in the CP direction the initial transients generated a fast rise but were spatiotemporally spread out over time so did not sum to a large peak in the response at the distal dendrite recording site. This effect tended to increase DSI (0.15). However, at the same 200 *μ*m/s velocity in models with only midget bipolars and a 200 *μ*m bar (Fig. 6F, brown traces), in the CP direction the secondary transient from distal bipolar inputs summed with the delayed initial transients from proximal bipolars to make a delayed peak at the distal recording site, which tended to decrease DSI (0.08).

At 5000 *μ*m/s, the stimulus bar evoked only a single initial transient in midget bipolars due to their RF surround delay and biophysical properties, and the resulting initial transients along a ~100 *μ*m starburst dendrite were evoked over ~20 ms. In the CF direction, the spatiotemporal and electrotonic propagation delays temporally compressed and summed the initial transients at the distal recording site, but in the CP direction, the spatiotemporal and propagation delays temporally spread out the initial transients. These two mechanisms generated different effects for responses evoked by 500 *μ*m and 200 *μ*m bars. With a 500 *μ*m bar (Fig. 6I) moving in the CF direction, the midget bipolar response (brown traces) was ~100 ms long due to the width of the bar, evoking a flat-topped response at the distal recording site. In the CP direction, the initial transient from the 500 *μ*m bar generated a fast initial rise at the distal recording site, but even with propagation delays the 100 ms response duration evoked by the width of the bar summed to generate a peak nearly equal in amplitude to the CF response, which tended to decrease DSI (0.04). With a 200 *μ*m bar, the midget bipolar responses were ~40 ms due to the width of the bar, and they summed with compression due to electrotonic propagation to generate a similar amplitude in the CF direction compared to the CF peak from the 500 *μ*m bar (Fig. 6G). However, in the CP direction the initial transient evoked a fast rise, but the narrower bipolar response was spread out due to spatiotemporal and propagation delays, and so did not sum to generate a peak as large as the CF response. This reduced the CP response amplitude and increased the directional difference and DSI (0.22).

### Effect of electrotonic summation on the dendritic DS

**Figure 9.**
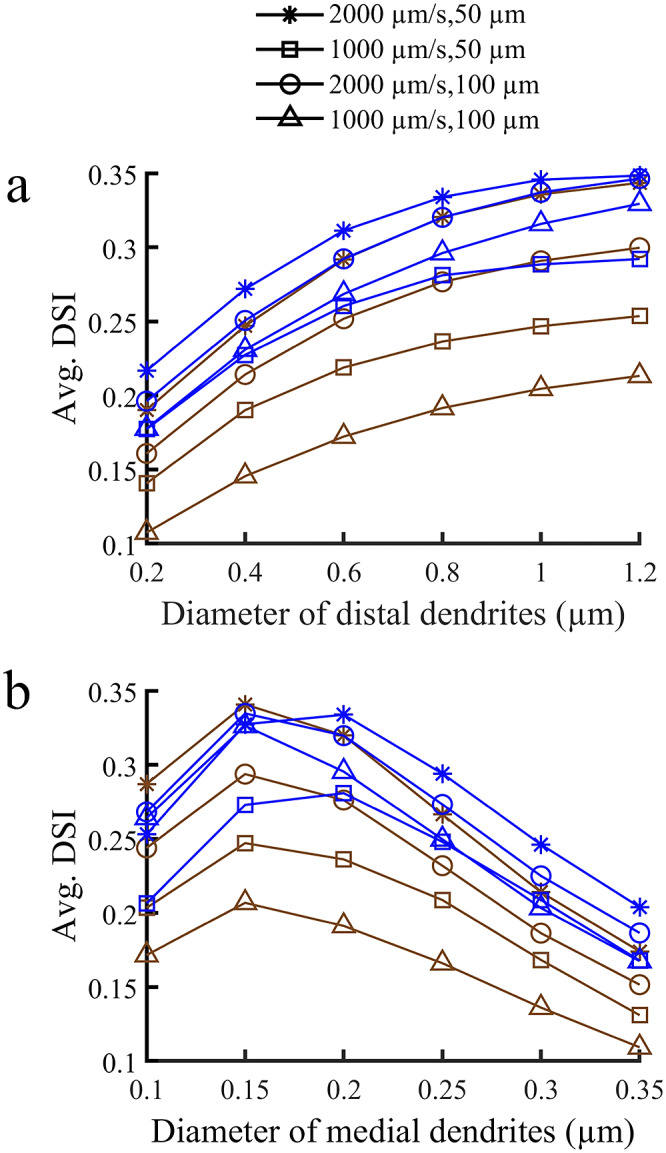
Effect of starburst dendritic diameter on the two mechanisms for DS. Plots illustrate the effect of varying distal (A) and medial (B) dendritic diameter on average DSI. Brown traces, only midget bipolar inputs; Blue traces, model with both midget and DB4/5 bipolars. The starburst dendritic voltage responses were evoked under different bar stimuli (velocities 1000 and 2000 *μ*m/s; bar width 50 and 100 *μ*m) and a mean DSI was computed from 30 runs of each model. The DSI reaches maximum for both models when the medial dendritic diameters are between 0.2 and 0.25 *μ*m, and the distal dendritic diameters are greater than or equal to 0.8 *μ*m.

The DS generated in the starburst dendrites was affected by the EPSP propagation speed and spatial extent of electrotonic summation, which are affected by the dendritic diameter, length, and biophysical properties (axial and membrane resistance, and membrane capacitance). To investigate the sensitivity of the model starburst to varying the medial and distal dendritic diameters on DS, we measured the distal dendritic voltage responses evoked by different bar stimulation conditions (velocities = 1000 and 2000 *μ*m/s; bar widths = 50 and 100 *μ*m). Following the original realistic model (see Materials and methods), we created models with incremental changes in their distal and medial dendrite diameters (Fig. 9A,B). As the distal dendrite diameters were increased in a series of models from 0.2 to 1.2 *μ*m with an increment of 0.2 *μ*m, the DSI increased for diameters up to 0.8 *μ*m, but not much more for larger diameters (Fig. 9A), which were larger than the best-fitted charging curve result (0.4 *μ*m). This implied that the exact distal dendritic diameter does not much affect the DS of starburst dendrites. When the medial dendrite diameter in another series of models was increased from 0.1 to 0.35 *μ*m with an increment of 0.05 *μ*m, the DSI reached a maximum when the medial dendritic diameters were between 0.2 and 0.25 *μ*m (Fig. 9B), consistent with the best-fitted charging curve result (0.2 *μ*m), and similar to the measured medial diameter (0.15–0.2 *μ*m, inset of Fig. 2). The addition of DB4/5 bipolar inputs increased the overall DSI but did not change the results from different dendrite diameters on DSI, implying that the diameters of starburst dendrites mainly affect the morphological mechanism. The amplitude and waveshape of EPSPs at different locations in the relatively thick distal dendrites were similar, implying that the precise location of the recording point in the distal dendrites was not critical.

### Calcium channels in starburst dendrites

**Figure 10.**
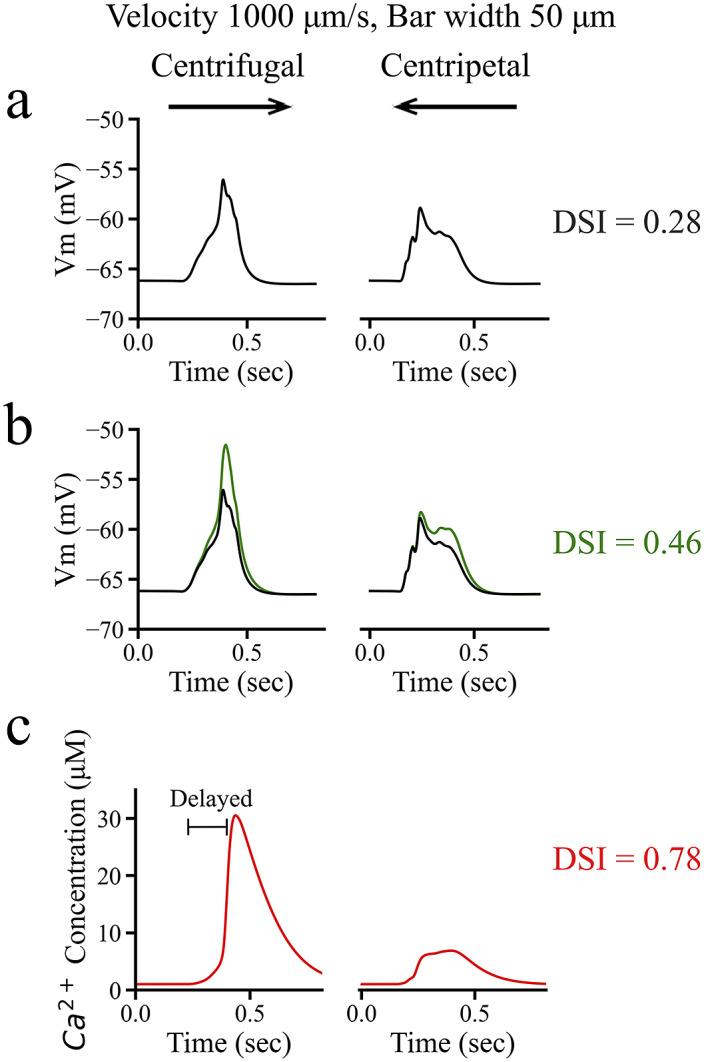
Calcium channels in starburst dendrites can enhance the DS initiated by the morphological and space–time mechanisms. Plots of distal starburst dendritic voltage and calcium responses (at purple asterisk from Fig. 3) evoked by a bar of 50 *μ*m width moving at a velocity of 1000 *μ*m/s in a representative example model that includes both midget and DB4/5 bipolar cells. (A) Plot of distal dendritic voltage response showing that the model generates modest DS without calcium channels (DSI = 0.28). (B) Plot of voltage response for the model with slowly inactivating (N/P/Q type) calcium channels added, in which the densities of calcium channels were 0.2 mS/cm^2^ at the soma and proximal dendrites, 3.5 mS/cm^2^ at medial dendrites and 7.5 mS/cm^2^ at distal dendrites. The calcium channel currents amplified the EPSPs and enhanced the DSI from (A) (black) to a DSI of 0.46 (green). Note that the peak of the green trace is superimposed upon the original peak of the black trace. (C) Plot of calcium concentration (DSI = 0.78, red) in the same model as the green voltage trace in (B). Notably, the calcium concentration bursted with a time delay of ~150 ms due to a voltage threshold of calcium channels around −55 mV. The DSI values in this figure were derived from one model; overall the average DSI for the calcium transient was a small fraction of this value because the highly nonlinear calcium activation effectively amplified variability in EPSP amplitude (see section “Discussion”).

Measurements by Kim et al. (2022) of direction-selective calcium responses from macaque starburst dendrites showed a greater DSI than we found in models in which the dendritic calcium channels were omitted (Figs. 5–9). The calcium channels can enhance the DS at the tip of starburst dendrites initiated by the morphological mechanism (Tukker et al., 2004). To investigate how much the calcium channels can enhance the DS from both morphological and space–time mechanisms, we analyzed a representative example model with and without calcium channels, stimulating with a bar of 50 *μ*m moving at a velocity of 1000 *μ*m/s (Fig. 10A,B). Slowly inactivating (N/P/Q type) calcium channels were added to the model (see Fig. 10B caption). As an example of a typical response, we adjusted the stimulus amplitude so that the EPSP amplitude in the CF direction was supra-threshold, and in the CP direction was subthreshold. With the calcium channel densities present, the DSI of the starburst dendritic voltage responses in this example was increased from 0.28 to 0.46 (Fig. 10B), and the DSI of the dendritic calcium response increased further to 0.78 (Fig. 10C). Notably, activation of the calcium channels was highly nonlinear and regenerative, in which the calcium currents activated by the synaptic inputs amplified the EPSP. The calcium concentration rose rapidly with a time delay of ~150 ms due to a voltage threshold for calcium channel activation around −55 mV. The duration of the calcium event depended on the calcium pump rate, but this had little effect on DS. Although this example showed that calcium channels can greatly amplify the directional difference, the average DSI computed from the set of 30 random starburst rotations and bipolar cell locations did not reflect the same degree of amplification, because the amplitude of the calcium transients varied widely due to the highly nonlinear activation of the calcium channels. The reason was that without several important gain control mechanisms that control subthreshold EPSP amplitude, the amplitude of the calcium transients was not well regulated (see section “Rationale for the specific model”). For example, in some cases, EPSP amplitude in CF and CP directions was above threshold and generated calcium transients in both directions, and in other cases, EPSP amplitude was below threshold in both directions and did not generate calcium events. In these cases, the calcium nonlinearity did not amplify the DSI. These results highlight the need for more complete models of the starburst biophysics and its network connectivity.

### Comparing DSI in macaque and mouse

In order to derive more intuition about the effect of morphology and connectomics on the two mechanisms for DS, we ran models using the starburst morphology and bipolar connectivity from the mouse retina (Ding et al., 2016). The mouse starburst cell is similar in diameter to the macaque starburst cell, but bipolar cell inputs are limited to the inner 2/3 of the dendritic tree. Another difference between the mouse and macaque starburst is that the density of bipolar inputs in the mouse is much higher, so the model of the mouse starburst received ~340 bipolar inputs (Fig. 4). The relative densities of sustained and transient bipolars were similar in the mouse model but the transient bipolars extended closer to the soma. The mouse model was run for 30 instances of random rotations of the starburst and random placement of the bipolar cells, using the same methods as for the macaque model. The results of DSI computed from the mouse models were similar to the macaque (Table 2). At high velocities, the morphological mechanism provided the largest part of DSI, and at low velocities with large objects, the space–time mechanism dominated DSI. Neither the density of bipolar cells nor their positioning limited to the inner 2/3 of the dendritic tree appeared to be critical for the key computation by the two mechanisms for DS. This further supported the findings from the macaque model, that the electrotonic properties of the dendrites and the different spatial locations of sustained *versus* transient bipolar cell inputs are sufficient to specify the stimulus dependence of DSI at starburst dendritic tips.

**Table 2.** Summary of the average DSI values from models of macaque and mouse starburst amacrine cells evoked by different velocities (μm/s) and bar widths (μm) for sustained and for sustained + transient bipolar cell inputs

Stimulus velocity	Bar width	Bipolar type	DSI macaque average	s.e.	*P*-value	Signif.	DSI mouse average	s.e.	*P*-value	Signif
100	50	Sustained	0.005	0.006	1.0 × 10^−3^	***	0.014	0.003	2.3 × 10^−7^	***
		Sustained + transient	0.096	0.027			0.122	0.017		
200	50	Sustained	0.039	0.007	7.0 × 10^−4^	***	0.0577	0.005	2.6 × 10^−8^	***
		Sustained + transient	0.125	0.024			0.166	0.016		
500	50	Sustained	0.127	0.010	4.8 × 10^−6^	***	0.159	0.009	5.4 × 10^−10^	***
		Sustained + transient	0.215	0.019			0.260	0.015		
1000	50	Sustained	0.236	0.013	8.9 × 10^−5^	***	0.257	0.010	7.4 × 10^−7^	***
		Sustained + transient	0.281	0.014			0.296	0.012		
2000	50	Sustained	0.320	0.013	0.097		0.309	0.008	2.0 × 10^−4^	
		Sustained + transient	0.334	0.009			0.288	0.009		
5000	50	Sustained	0.283	0.010	0.84		0.243	0.008	1.5 × 10^−5^	
		Sustained + transient	0.282	0.010			0.205	0.006		
10,000	50	Sustained	0.177	0.008	0.13		0.146	0.008	4.1 × 10^−3^	
		Sustained + transient	0.188	0.009			0.124	0.004		
100	100	Sustained	0.042	0.009	1.6 × 10^−8^	***	0.079	0.008	2.7 × 10^−10^	***
		Sustained + transient	0.179	0.017			0.203	0.014		
200	100	Sustained	0.055	0.005	1.3 × 10^−9^	***	0.085	0.005	1.8 × 10^−14^	***
		Sustained + transient	0.194	0.017			0.232	0.013		
500	100	Sustained	0.110	0.007	2.7 × 10^−12^	***	0.135	0.004	1.1 × 10^−15^	***
		Sustained + transient	0.254	0.016			0.309	0.012		
1000	100	Sustained	0.192	0.011	9.1 × 10^−13^	***	0.204	0.006	9.0 × 10^−17^	***
		Sustained + transient	0.296	0.014			0.337	0.010		
2000	100	Sustained	0.277	0.010	9.6 × 10^−8^	***	0.256	0.006	1.2 × 10^−11^	***
		Sustained + transient	0.320	0.011			0.300	0.007		
5000	100	Sustained	0.274	0.009	0.33		0.224	0.006	1.5 × 10^−5^	
		Sustained + transient	0.278	0.009			0.205	0.005		
10,000	100	Sustained	0.176	0.007	0.280		0.135	0.006	5.3 × 10^−5^	
		Sustained + transient	0.181	0.008			0.116	0.004		
100	200	Sustained	0.102	0.014	3.6 × 10^−6^	***	0.221	0.007	7.7 × 10^−8^	***
		Sustained + transient	0.195	0.020			0.312	0.014		
200	200	Sustained	0.082	0.009	4.6 × 10^−10^	***	0.183	0.008	1.7 × 10^−12^	***
		Sustained + transient	0.205	0.018			0.291	0.012		
500	200	Sustained	0.081	0.004	3.6 × 10^−12^	***	0.133	0.007	9.1 × 10^−17^	***
		Sustained + transient	0.233	0.015			0.298	0.013		
1000	200	Sustained	0.114	0.005	2.8 × 10^−12^	***	0.127	0.006	3.5 × 10^−18^	***
		Sustained + transient	0.259	0.013			0.314	0.013		
2000	200	Sustained	0.165	0.007	1.8 × 10^−12^	***	0.139	0.005	1.0 × 10^−18^	***
		Sustained + transient	0.280	0.012			0.291	0.010		
5000	200	Sustained	0.215	0.007	2.6 × 10^−11^	***	0.165	0.004	4.2 × 10^−9^	***
		Sustained + transient	0.269	0.009			0.194	0.005		
10,000	200	Sustained	0.180	0.007	0.81		0.128	0.004	6.1 × 10^−7^	
		Sustained + transient	0.180	0.007			0.116	0.004		
100	500	Sustained	0.159	0.014	7.6 × 10^−3^	***	0.237	0.007	3.2 × 10^−6^	***
		Sustained + transient	0.209	0.020			0.312	0.015		
200	500	Sustained	0.148	0.011	2.4 × 10^−5^	***	0.247	0.008	4.8 × 10^−8^	***
		Sustained + transient	0.220	0.017			0.322	0.014		
500	500	Sustained	0.134	0.008	7.1 × 10^−11^	***	0.235	0.010	1.1 × 10^−11^	***
		Sustained + transient	0.254	0.016			0.341	0.014		
1000	500	Sustained	0.115	0.006	6.1 × 10^−14^	***	0.190	0.009	3.6 × 10^−14^	***
		Sustained + transient	0.262	0.015			0.337	0.012		
2000	500	Sustained	0.079	0.004	1.7 × 10^−16^	***	0.119	0.007	1.0 × 10^−18^	***
		Sustained + transient	0.261	0.014			0.294	0.010		
5000	500	Sustained	0.038	0.002	2.2 × 10^−17^	***	0.053	0.004	5.1 × 10^−21^	***
		Sustained + transient	0.206	0.011			0.178	0.006		
10,000	500	Sustained	0.078	0.003	5.4 × 10^−13^	***	0.062	0.002	1.5 × 10^−11^	***
		Sustained + transient	0.138	0.007			0.099	0.003		

Standard error values for the mean DSI values are listed under “s.e.”. The statistical comparison (probability that there is no difference) between the average DSIs from sustained and sustained + transient models is shown in the “*P*-value” column. The cases in which the sustained + transient DSI was greater and had a significant (*P* < 0.01) difference are starred (***) in the “signif.” columns. The cases not starred are all for the higher velocities (2000, 5000, 10,000 *μ*m/s) in which the morphological mechanism represents the major component of DS.

## Discussion

The simulations suggest that both morphological and space–time mechanisms can contribute to directional responses in macaque starburst amacrine cell dendrites, and that their relative contributions are dependent upon the spatial extent and velocity of the stimulus. At low velocities with large object sizes, the models containing DB4/5 transient bipolar cells produced DSI values significantly different than the models containing only midget bipolar cells (Table 2). At high velocities with small object sizes the models containing DB4/5 transient bipolar cells produced DSI values that converged with those of the models containing only midget bipolar cells (Fig. 8).

The “morphological mechanism” in these models contributed the largest component of DS with small visual objects (high spatial frequency) at high velocities. The “space–time mechanism” depended on summation of large objects (low spatial frequencies) and made the largest contribution at low velocities which can evoke relatively sustained responses from midget bipolars. The space–time mechanism relied mainly on the differences in response kinetics and spatial location between sustained and transient cell types. By contrast, the morphological mechanism relied on the waveshape-filtering properties of the dendrites. Therefore, both mechanisms are affected by the visual object size and velocity.

The morphological mechanism for DS depends upon electrotonic propagation and spatiotemporal summation of EPSPs evoked by bipolar inputs spaced along the extremely fine radial dendrites. This mechanism is defined by the cell’s morphology, including the dendritic tree connected to the soma, the branching pattern of the dendrites, and the diameter of different sections of the dendrites from soma to the medial and distal regions. The morphological mechanism produces maximum DS when the distal dendrite is partially isolated from the soma (Tukker et al., 2004). This partial electrotonic isolation causes EPSPs from proximal bipolar cells to be delayed as they propagate centrifugally toward the dendritic tips. The larger dendritic diameter of the distal section enhances the delay, because the capacitance of the thicker tips in series with the axial resistance of the thinner medial section produces a low-pass filter. The delay allows proximal and distal EPSPs evoked by centrifugal motion to overlap and temporally sum at the distal tips. This temporal summation effect causes the directional difference observed between centrifugal and centripetal motion when the delay along the length of the dendrite is equivalent to the stimulus velocity.

Another component of the morphological mechanism is the “sealed cable” effect (Vlasits et al., 2016) in which synaptic loci near the dendritic tips have higher input resistance than those near the soma. Because of this effect, excitatory synaptic conductances near the dendritic tip tend to cause larger depolarizations than similar conductances near the soma. The distal bipolar cell inputs to the thicker distal dendrites (0.4–0.8 *μ*m) are affected by the sealed cable effect, because the very thin (0.2 *μ*m) medial dendrites increase the series resistance to the soma and thus the electrotonic isolation (Fig. 2). At the distal recording site, in the centrifugal direction this effect tends to enhance peak response amplitude by increasing the amplitude of distal EPSPs summed with lagged proximal EPSPs, and in the centripetal direction tends to generate a quickly rising EPSP due to locally summed distal inputs without enhancing the peak response amplitude. This will boost DSI due to both the morphological and space–time mechanisms. These effects are apparent in the voltage recordings (Fig. 6).

The “space–time” mechanism for DS implemented in this study depends upon summation of two types of bipolar cell responses (Kim, 2014): a sustained response from midget bipolar cells, and a more transient response from DB4/5 bipolar cells. In response to a large visual object moving away from the soma (centrifugal), the proximal dendrites receive a prolonged EPSP, so that the EPSP continues while the object moves toward the distal dendrite (Fig. 5A,B). At the distal dendrite, EPSPs from transient bipolar cells (DB4/5) efficiently sum with the EPSP from more proximal bipolar cells (Fig. 5B). In the opposite (centripetal) direction, the transient EPSP arrives first, but then decays before the more sustained proximal EPSPs can sum with it (Fig. 5B). This mechanism functions best for visual objects that span the radial length of the dendrites or more, so that the sustained and transient EPSPs overlap and efficiently sum in the distal dendrite. At low velocities, its contribution to DS is greater compared to the morphological mechanism which only functions efficiently at high velocities (Fig. 8 and Table 2). The space–time mechanism is also dependent upon the amplitude and duration of the transient, and the number of transient bipolar cells that provide input to a starburst dendrite.

For velocities of 500 *μ*m/s or below, the DSI generated by the space–time mechanism reaches maximum when the stimulus size is about equal to the radius of the starburst dendritic arbor (i.e., the span of a dendrite) (Fig. 8F). The reason is that for the optimal efficiency the space–time mechanism requires both the proximal sustained and distal transient bipolar inputs to overlap in time. Larger stimuli do not much increase the efficiency because the distal transient has decayed before the stimulus terminates. In our model, the space–time mechanism with its transient input from DB4/5 bipolars also depends at higher velocities (1000 *μ*m/s and above) on the transient input amplitude. The reason is that at higher velocities the transient input engages the morphological mechanism, so the greater amplitude of the transient input (Fig. 5D,H) when summed with the input from midget bipolars enhances DSI (Fig. 6D,E,G,I). However, the maximum DSI also depends upon stimulus velocity (Figs. 6 and 8), because the morphological mechanism functions optimally for smaller stimuli and faster velocities (Figs. 6 and 7). Therefore, the optimal directional difference with both mechanisms together is a trade-off between stimulus size and speed (Figs. 6–8).

### Comparison with mouse and rabbit

The morphology and connectomics of the macaque starburst amacrine modeled here are qualitatively similar to starbursts of mouse and rabbit, but differ sufficiently to justify an additional modeling effort. The macaque starburst dendrites receive bipolar cell synaptic inputs from proximal to distal regions, similar to rabbit (Famiglietti, 1991), but differ from mouse in which the bipolar inputs are limited to the inner radial 2/3 of the dendrite extent (Ding et al., 2016; Vlasits et al., 2016). However, the computational model used by Ding et al. (2016) only included one type of bipolar cell. A potentially salient difference is that in mouse, several types of bipolar cell make contact onto starburst dendrites, and the density of mouse bipolar contacts is several-fold greater than in macaque. Also, in the macaque starburst distal regions, about 50% of the bipolar inputs are from transient DB4/5 bipolars, which is similar to the situation in mouse in which inputs from several transient bipolar types contact starburst dendrites in the distal region out to the 2/3 radial limit. These differences in connectivity did not much affect the function of the two mechanisms for DS explored in our models. As shown in Table 2, our models of the mouse starburst gave similar DSI values to the macaque models.

As pointed out by Ding et al. (2016), the mouse eye is smaller than the eye of rabbit and the macaque studied here, and this difference in size is reflected in stimulus velocities being 5 to 7-fold lower at the mouse retina. Yet, starburst dendritic arbor diameter (200–250 *μ*m) is similar in these species. The circuitry of the starburst amacrine cell network in mouse has adapted to this lower velocity by evolving several features that provide sensitivity to these lower velocities. Neighboring mouse starburst amacrines overlap to a greater extent compared with rabbit and macaque, and they make inhibitory contacts on proximal dendrites close to their neighbors’ somas (Ding et al., 2016). The proximal inhibition along with the lack of distal bipolar input is thought to assist and enhance the directional difference of EPSPs in mouse starburst dendrites at low velocities (Ding et al., 2016; Vlasits et al., 2016). In comparison, rabbit starbursts are thought to provide inhibition to neighbor starbursts in more distal dendritic regions (Lee & Zhou, 2006; Taylor & Smith, 2012).

### Rationale for the specific model

The model of the macaque starburst amacrine cell developed here has several obvious omissions. It omits mechanisms for adaptation and other signal processing features that are known to exist in the presynaptic circuitry, and other signal-processing mechanisms in the starburst network. For example, photoreceptors and their multiple adaptational mechanisms for background level and contrast were omitted. Instead, the stimulus was presented directly into the bipolar cells presynaptic to the starburst cell *via* optical blur functions (Kim et al., 2022). Other omissions were putative network mechanisms such as cholinergic feedback, and GABAergic and glycinergic inhibition. Further, voltage-gated calcium channels and sodium channels (see Fig. 1) were omitted from the starburst amacrine cell in the model for comparisons between the morphological and space–time mechanisms (Figs. 5–9). Although inclusion of these mechanisms might allow the model to be more realistic, and would be worthwhile to pursue in future work, they are unnecessary for the comparison of the two mechanisms for DS considered here, and their omission reduced the number of parameters that would require calibration.

Although calcium channels are known to exist in starburst dendrites and are necessary for neurotransmitter release from varicosities near the distal tips, we chose to omit them for several reasons. In mouse and rabbit, several other mechanisms contingent upon the local circuitry are thought to modulate the amplitude of directional signals in starburst amacrine dendrites, and similar mechanisms may also exist in macaque. For example, release of acetylcholine by starburst dendrites in mouse retina activates depolarizing receptors in type 5 and type 7 bipolar cells that provide excitatory inputs to starburst dendrites, which may generate positive feed-forward input to neighbor starbursts (Hellmer et al., 2021). Calcium channel activation in starburst dendrites can be modulated by mGluR2 signaling (Koren et al., 2017). GABA release by starburst dendrites inhibits neighboring starburst cells in mouse and rabbit, generating positive feedback and disinhibition (Lee & Zhou, 2006; Chen et al., 2016; Ding et al., 2016; Morrie & Feller, 2018; Chen et al., 2020). Glycine released by narrow-field amacrine cells in mouse retina is a potent inhibitory modulator of starburst EPSP amplitude and contrast gain (Jain et al., 2022). Further, the nonlinear voltage activation of calcium channels in starburst dendrites can modify the DS for neurotransmitter release (Tukker et al., 2004), and calcium channel activation can regeneratively amplify the directional EPSP signals (Fig. 10B). The presence of voltage-gated sodium channels is likely to enhance the nonlinearity of the threshold for generation of calcium transients.

Although all these mechanisms can modulate the gain and peak amplitude of subthreshold EPSPs and therefore modulate the likelihood of calcium events and neurotransmitter release in starburst dendrites, to include them in the model would necessarily detract from the comparison of the two mechanisms for DS investigated here. The reason is that the origin of the DS asymmetry must be in the subthreshold EPSP, and must derive from the kinetic properties of the inputs and the electrotonic cable properties of the dendrites. The comparison between the two mechanisms that we make here attempts to isolate those kinetic and electrotonic summation properties. Voltage-gated calcium and sodium channels are highly sensitive to subthreshold EPSP amplitude, and the directional differences in this subthreshold amplitude are predicted by the two mechanisms for DS included in the model without the other gain-modulating mechanisms.

### Relation to behavioral performance

Our results from both the mouse and macaque models predict that the space–time mechanism that includes transient bipolar cell inputs at distal dendritic locations can enhance directional differences evoked by large, slow-moving stimuli more effectively than the morphological mechanism. Due to the ~7-fold smaller size of the mouse eye (1 deg = 30 *μ*m for mouse *versus* 1 deg = 200 *μ*m for macaque) and, since both species have starburst amacrine cells of roughly equal size, the mouse starburst space–time mechanism is tuned for visual stimuli ~7-fold larger than for macaque, moving at ~7-fold higher velocities, defined in degrees of visual angle. Thus, a visual object 1 deg in size moving at 1 deg/s travels at 200 *μ*m/s across the macaque retina to efficiently engage the space–time mechanism in the macaque retina, but the same movement and engagement of the space–time mechanism in the mouse retina is evoked by a 6.6-fold larger object moving at 6.6 deg/s. An object moving at 10 deg/s travels at ~2000 *μ*m/s across the macaque retina, which produces near-optimal DSI due to the morphological mechanism. Yet the same ~2000 *μ*m/s movement across the mouse retina is evoked by an object moving at 66 deg/s. This might be important for the mouse in attending to fast-moving objects such as predators.

Studies of velocity tuning in the human and nonhuman primate visual system indicate sensitivity to speeds in the range of 0.1 deg/s to greater than 60 deg/s (McKee & Nakayama, 1984; De Bruyn & Orban, 1988; Nover et al., 2005). This range corresponds well to the range of velocities employed for simulations in this study (Fig. 8). Studies of velocity tuning in the mouse indicate sensitivity to speeds in visual space in the same range as humans and nonhuman primates (Weng et al., 2005; Umino et al., 2008; Gao et al., 2010). Yet, as explained above, the velocity sensitivity of the two mechanisms for DS in the mouse starburst in terms of visual angle is roughly sevenfold higher than for the macaque retina. However, additional network mechanisms involving the mouse starburst are thought to enhance its sensitivity to low velocities (Ding et al., 2016). Thus, the range of velocities appropriate for mouse appears to be well covered by our simulations and it is reasonable to suppose that the conclusions that we have reached about the contribution of the morphological and space–time mechanisms to retinal coding of direction are relevant for species as diverse as mouse and the macaque.

## Footnotes

1 DB4 and DB5 are diffuse bipolar cells whose axons arborize in stratum 3 of the inner plexiform layer (Tsukamoto & Omi, 2016). Kim et al. (2022) found one type of diffuse bipolar cell that created a single largely nonoverlapping mosaic of axonal spread in stratum 3 that connects to the starburst cell. It is referred to here as DB4/5 since it remains unclear from that study whether this cell type should be called DB4 or DB5.
